# Association Between E-cigarette Use and Asthma Exacerbations Using Nationally Representative Survey Data

**DOI:** 10.7759/cureus.75498

**Published:** 2024-12-10

**Authors:** Felicia P Tanu, Mario F Perez, Tucker V Rathe, Eric M Mortensen

**Affiliations:** 1 Internal Medicine, University of Connecticut School of Medicine, Farmington, USA; 2 Pulmonary/Critical Care, University of Connecticut Health, Farmington, USA; 3 Internal Medicine, University of Connecticut Health, Farmington, USA; 4 Internal Medicine, VA North Texas Health Care System, Dallas, USA

**Keywords:** acbs, asthma, asthma exacerbations, brfss, cigarette, cigarette smoking, e-cigarette smoking, nicotine, vaping, young adult

## Abstract

Introduction

Despite limited knowledge of its potential health effects, electronic cigarette (e-cigarette) use has become increasingly popular in the United States (US). Cigarette smoking is linked to a higher risk of asthma, and e-cigarettes may have similar effects. This study’s aim was to examine the association between e-cigarette use and asthma exacerbations in US adults with known asthma.

Methods

The 2018 Asthma Call Back Survey (ACBS), paired with corresponding data from the 2018 Behavioral Risk Factor Surveillance System (BRFSS), was utilized for this analysis. Individuals were divided into the following categories: exclusive e-cigarette users, exclusive cigarette users, dual e-cigarette and cigarette users, and never users. A multivariable logistic regression model utilizing a complex survey design was completed after applying weights for population representation and adjusting for potential confounders.

Results

Of the 11,598 adults in the study, 453 (3.9%) were exclusive e-cigarette users, 3,735 (32.2%) were exclusive cigarette users, 1,484 (12.8%) were dual users, and 5,926 (51.1%) were never users. There was no statistically significant association between asthma exacerbations and exclusive e-cigarette use (OR: 0.88; 95% CI: 0.56-1.38), exclusive cigarette use (OR: 0.80; 95% CI: 0.60-1.05), or dual-use (OR: 0.98; 95% CI: 0.68-1.41).

Conclusion

There was no association among the categories of exclusive e-cigarette users, exclusive cigarette users, and dual users with asthma exacerbations among adults 18+ with asthma. The results emphasize a need to continue e-cigarette use research to assess future health effects and formulate appropriate public health recommendations.

## Introduction

Electronic cigarette (e-cigarette) use has been increasing over the past several years, which is a potential public health concern due to limited known information regarding possible both short- and long-term health effects. In 2021, 4.5% of United States (US) adults 18+ were current e-cigarette users compared to 3.2% of adults in 2018 [1,2]. E-cigarettes generate an aerosol by heating a liquid that frequently contains nicotine, which may affect the young adult brain and fetal development, and other chemicals, such as solvents, flavorings agents, tetrahydrocannabinol, and cannabidiol [3]. It is known that cigarette smoking is associated with an increased risk of developing asthma and can lead to poorer control of symptoms in individuals with known asthma [4]. It is suggested that e-cigarette use similarly increases the risk of lung diseases as they lead to similar inflammatory responses seen in conventional cigarettes. For example, the inflammatory biomarker chitinase-like protein YKL-40 has been shown to be elevated in the sputum and serum of cigarette smokers and individuals with chronic obstructive lung disease (COPD), as well as the serum of e-cigarette users without a prior history of pulmonary disease [5,6].

Asthma is a chronic inflammatory disease of the airways characterized by recurrent episodes of shortness of breath, wheezing, and cough that, if left untreated, may be life-threatening or lead to chronic airway remodeling [7]. According to the Centers for Disease Control and Prevention (CDC), 7.7% of US adults lived with asthma in 2018 [8]. Currently, limited data on the health outcomes of e-cigarette use in asthma patients are available. A report from the National Academies of Science, Engineering, and Medicine on the Public Health Consequences of E-cigarettes suggests that e-cigarettes increase wheezing, coughing, and asthma exacerbations in adolescents [9]. Perez et al. concluded that adult e-cigarette users who never smoked cigarettes had a higher risk of reporting a diagnosis of asthma compared to those who did not utilize e-cigarettes [10].

Conversely, there are other reports, such as Polosa et al. [11], that suggest that e-cigarettes might help patients with asthma reduce their cigarette consumption and reduce asthmatic symptoms. The United Kingdom’s Royal College of Physicians advocates for e-cigarette use in smoking cessation efforts and even asserts that they are safer than traditional cigarettes [12]. However, the US Preventive Service Task Force has reported insufficient data to conclude the effectiveness of using e-cigarettes in assisting attempts to quit [13]. In addition, the long-term effects of e-cigarettes are currently unknown, but in 2019, the CDC reported that e-cigarette use was associated with lung injury and death among adults, a condition currently known as e-cigarette use-associated lung injury [2].

This study examines the association between exclusive e-cigarette use, exclusive cigarette use, and dual e-cigarette and cigarette use with asthma exacerbations in US adults with known asthma. Data from the 2018 Asthma Call Back Survey (ACBS) and corresponding data from the Behavioral Risk Factor Surveillance System (BRFSS) were utilized for qualitative secondary data analysis. Our priori hypothesis is that exclusive e-cigarette use in patients diagnosed with asthma will be associated with increased asthma exacerbations after adjusting for potential confounders compared to individuals who never smoked.

This article was previously presented as a meeting abstract at the Medical & Dental Research Day at the University of Connecticut School of Medicine on February 23, 2024, and at the Society of General Internal Medicine National Meeting on May 16, 2024.

## Materials and methods

Study sample

The 2018 ACBS, which contains data on e-cigarette use behavior, paired with corresponding data from the 2018 BRFSS, was used for this cross-sectional study. The BRFSS is a health-related survey conducted by the CDC. Study participants were selected using a random digit dialing technique on landlines and cell phones. They were asked a standardized core questionnaire, optional modules, and state-added questions [14,15] (see Appendix Table 5). ACBS is a survey developed by the Asthma and Community Health Branch of the National Center for Environmental Health. Individuals who previously reported a history of asthma during the BRFSS were contacted via telephone two weeks later for a more in-depth interview on asthma control and related behaviors for inclusion in the ACBS [16,17] (see Appendix Table 6). Participants were asked various questions pertaining to their asthma, such as medication use, insurance coverage, and healthcare utilization. Both the BRFSS and ACBS contain public de-identified data and, as such, do not require IRB approval.

In this study’s regression model, individuals who responded yes to the question “Ever told you have chronic obstructive pulmonary disease, emphysema, or chronic bronchitis?” were excluded. For the outcome variables of every asthma attack and for any asthmatic symptoms in the past 30 days, individuals who responded with “don’t know” or “refused” were dropped from the study.

Measures

Data were collected based on sex, race/ethnicity, educational level, BMI, employment, income, alcohol consumption, influenza vaccination, insurance status, and past medical history. A binary sex categorization of male/female was utilized in this study and was evaluated through the questions “What is your sex?” or “What was your sex at birth?”. Participants were considered to currently have asthma through the confirmation questions “Have you ever been told by a doctor, nurse, or other health professionals that you had asthma?” and “Do you still have asthma?”. E-cigarette use was assessed with the question “Have you ever used an e-cigarette or other electronic vaping product, even just one time, in your entire life?”. Never e-cigarette users were recorded with a “no”, while ever e-cigarette users were recorded with a “yes”. Individuals who solely vaped e-cigarettes and did not use cigarettes were then isolated. These individuals were classified as “exclusive e-cigarette users”. Cigarette use was evaluated through the question “Have you smoked at least 100 cigarettes in your entire life?”. Similar to e-cigarette use, never-cigarette users reported “no”, while ever cigarette users reported “yes”. Afterward, those individuals who solely smoked cigarettes and did not use e-cigarettes were isolated. These individuals were classified as “exclusive cigarette smokers". Dual e-cigarette and cigarette use participants were defined as individuals who responded “yes” to both the e-cigarette and cigarette questions. Never smokers were defined as those who responded “no” to both the e-cigarette and cigarette questions.

All individuals who participated in the ACBS were asked further questions regarding their asthma history. To evaluate for any asthma attacks, individuals were asked to answer “yes” or “no” to: “During the past 12 months, have you had an episode of asthma or an asthma attack?”. The presence of asthmatic symptoms in the past 30 days was evaluated through the question “During the past 30 days, on how many days did you have any symptoms of asthma?”. Participants who reported any day of symptoms within the past 30 days were recorded as a “yes”, while those who reported no symptoms were recorded as “no”.

Individuals were divided into the following categories: exclusive e-cigarette users, exclusive cigarette users, dual e-cigarette and cigarette users, and never-smokers.

Statistical analysis 

We used data from the 2018 BRFSS/ACBS for secondary data analysis, which contained a total of 11,598 adults with asthma. In total, there were 453 participants who exclusively smoked e-cigarettes. We presented categorical variables as percentages and used 2 x 2 tables to compare among groups. Multivariable logistic regression models employing the complex survey design to assess the primary outcome of the association between smoking status and asthma exacerbations were used after controlling for confounders. Survey weights were included in the analysis to account for the loss of samples, which included non-respondents and those who did not wish to be called back between the BRFSS interview and the ACBS interview [18]. Results in this study are based on weighted data. All analyses were conducted utilizing Stata 18 (StataCorp LCC, College Station, TX). A two-tailed p-value < 0.05 was considered statistically significant.

## Results

Participant characteristics, healthcare utilization, and medication use by smoking status

In the 2018 BRFSS-ACBS, 11,598 adults (weighted N = 25,061,891) met this study’s inclusion criteria. Of those participants, 453 (3.9%) were exclusive e-cigarette users, 3,735 (32.2%) were exclusive cigarette users, 1,484 (12.8%) were dual users, and 5,926 (51.1%) were never users of either product. Descriptive statistics, including demographics and past medical history, of the study population are shown in Table 1. Exclusive e-cigarette users were more likely to be male (53.9%), aged 18-24 years (83.7%), and White users (50.0%). Exclusive cigarette users were more likely to be female (55.2%), aged 45-54 (22.0%), and White users (71.6%). Dual users were more likely to be female (51.6%), aged 18-24 (39.0%), and White users (67.8%). Similar to dual users, never users were more likely to be female (66.3%), aged 18-24 (33.0%), and White users (63.7%).

**Table 1:** Demographics and past medical history of participants according to smoking status ^a ^AI/AN, American Indian/Alaskan Native ^b ^Heavy drinkers are defined as adult men having more than 14 drinks per week and adult women having more than 7 drinks per week ^c ^HS, high school ^d ^Non-obese: BMI < 30.0, Obese: BMI ≥ 30.0 ^e ^Respiratory disease defined as individuals who have been diagnosed with COPD, emphysema, or chronic bronchitis ^f ^Yrs, years

	Weighted N=25,061,891; n=15,598 (%)	Exclusive e-cigarette user, Weighted N= 1,838,585; n=453 (%)	Exclusive cigarette user, Weighted N= 6,706,556; n=3,735 (%)	Dual user, Weighted N= 3,690,931; n=1,484 (%)	Never user, Weighted N= 12,825,819; n=5,926 (%)
Sex					
Male	9,197,714 (36.7)	53.9	44.8	48.4	34.7
Female	15,864,177 (63.3)	46.1	55.2	51.6	66.3
Age Groups					
18-24 yrs^f^	3,977,322 (15.9)	83.7	13.8	39.0	33.0
25-34 yrs	2,583,881 (10.3)	5.0	14.8	18.7	15.4
35-44 yrs	3,819,432 (15.2)	6.1	19.2	20.0	17.3
45-54 yrs	5,977,261 (23.9)	3.7	22.0	15.3	16.0
55-65 yrs	5,669,000 (22.6)	1.3	17.8	5.2	12.7
65 yrs +	3,034,995 (12.1)	0.3	12.5	2.1	5.7
Race					
White	19,445,521 (77.6)	50.0	71.6	67.8	63.7
Black	1,451,084 (5.8)	16.9	10.9	8.5	12.1
Asian	353,373 (1.4)	3.9	1.6	1.6	4.0
AI/AN ^a^	528,806 (2.1)	0.3	1.2	2.7	1.0
Hispanic	2,175,372 (8.7)	24.9	11.9	15.4	16.9
Other race	1,107,736 (4.4)	4.2	2.8	4.1	2.4
Ethnicity					
Non-Hispanic	22,904,062 (91.4)	75.1	88.2	84.6	83.2
Hispanic	2,157,829 (8.6)	24.9	11.8	15.4	16.8
Educational Level	,				
HS or Less ^c^	7,420,826 (29.6)	40.2	43.0	50.5	26.7
Some College or Higher	17,641,065 (70.4)	59.8	57.1	49.5	73.3
Other Factors					
BMI^d^					
Non-obese	14,896,788 (59.4)	70.4	58.0	60.0	62.5
Obese	10,165,103 (40.6)	29.6	42.0	40.0	37.5
Employment					
Unemployed	14,084,783 (56.2)	39.2	59.2	44.2	45.2
Employed	10,977,108 (43.8)	60.8	40.9	55.8	54.9
Household Income					
< $75,000	18,350,317 (73.2)	68.7	75.1	82.4	64.6
≥ $75,000	6,711,574 (26.8)	31.2	25.0	17.6	35.4
Times Binge Drinking ^b^					
Non-heavy Drinker	23,746,141 (94.8)	96.3	92.4	86.9	96.6
Heavy Drinker	1,315,749 (5.3)	3.8	7.6	13.1	3.4
Influenza Vaccinated					
No	13,994,560 (55.8)	71.0	57.1	76.5	61.4
Yes	11,067,331 (44.2)	29.0	43.0	23.5	38.6
Insurance					
Uninsured	1,488,676 (5.9)	10.9	7.0	18.5	6.5
Insured	23,573,215 (94.1)	89.1	93.0	81.5	93.5
Comorbidities					
History of Respiratory Disease ^e^					
Yes	6,167,732 (24.6)	6.0	29.1	32.9	11.1
History of Heart Attack					
Yes	2,062,594 (8.2)	0.8	10.2	6.6	4.4
History of Depressive Disorder					
Yes	8,696,476 (34.7)	35.4	39.5	49.3	28.9

Asthma-related symptoms and healthcare utilization by smoking status are described in Table 2. Among study participants, dual users were the most likely to report having an asthma attack within the last 12 months (38.3%), while exclusive e-cigarette users were the least likely to have had an asthma attack (24.6%). Additionally, 11.5% of dual users reported ever having an ER or urgent care visit in the past 12 months in comparison to only 3.7% of exclusive e-cigarette users. Moreover, 54.2% of dual users reported experiencing symptoms of asthma in the past 30 days in comparison to 34.9% of e-cigarette users, which is the smallest group to have experienced symptoms. There is noted to be a minimal number of exclusive e-cigarette users (1.5%), exclusive cigarette users (3.0%), dual users (4.3%), and never users (2.4%) who were hospitalized in the past 12 months.

**Table 2:** Asthma-related healthcare use of participants according to smoking status

		Exclusive e-cigarette user, Weighted N= 1,838,585; n=453 (%)	Exclusive cigarette user, Weighted N= 6,706,556; n=3,735 (%)		Dual user, Weighted N= 3,690,931; n=1,484 (%)		Never user, Weighted N= 12,825,819; n=5,926 (%)		
Asthma Episode/Attack in the last 12 months									
No		75.4	63.5			61.8	66.8		
Yes		24.6	36.5			38.3	33.2		
ER or Urgent Care visit past 12 months									
No		96.3	90.3			88.5	91.9		
Yes		3.7	9.7			11.5	8.1		
Had Symptoms Last 30 days									
No		65.2	49.4			45.8	57.9		
Yes		34.9	50.6			54.2	42.1		
Last saw a Physician									
Within the past year		35.5	55.7			44.4	50.2		
1-3 years		22.1	15.8			14.8	17.5		
3-5 years		10.1	6.0			9.6	7.9		
More than 5 years		32.2	22.6			31.3	24.4		
Last took Medications									
Less than 1 day		12.4	34.2			25.7	24.3		
1-6 days		3.6	8.7			5.8	8.8		
1 week to 3 months		13.1	9.3			14.8	10.7		
3 months to 1 yrs		13.9	7.9			6.9	11.8		
1-5 yrs		12.9	12.9			14.2	15.0		
More than 5 years		44.3	27.0			32.5	29.5		
Hospitalization past 12 months									
No		98.5	97.0			95.7	97.6		
Yes		1.5	3.0			4.3	2.4		

In Table 3, asthma medication use by smoking status is described. Most e-cigarette users on medication were on a short-acting beta agonist (SABA) (21.9%), while almost no participants were on short-acting muscarinic antagonist (SAMA) (0.6%), oral corticosteroids (OCS) (0.3%), or SAMA/SABA (0.5%). Similarly, exclusive cigarette users on medication were on a SABA (36.8%), while almost no participants were on SAMA (0.6%), OCS (2.0%), or SAMA/SABA (0.8%). The majority of dual users on medication were on a SABA (37.1%), while almost no participants were on SAMA (2.2%), OCS (2.0%), or SAMA/SABA (0.2%). Lastly, the majority of never users on medications were on SABA (31.1%). There were a minimal amount of never users on SAMA (0.08%), OCS (1.9%), or SAMA/SABA (0.9%).

**Table 3:** Asthma medication use vs. smoking status ^a ^ICS, inhaled corticosteroid ^b ^LABA, long acting β agonist ^c ^LTRA, leukotriene receptor antagonist ^d ^OCS, oral corticosteroid ^e ^SABA, short acting β agonist ^f ^SAMA, short acting muscarinic antagonist

	Exclusive e-cigarette user, Weighted N= 1,838,585; n=453 (%)	Exclusive cigarette user, Weighted N= 6,706,556; n=3,735 (%)	Dual user, Weighted N= 3,690,931; n=1,484 (%)	Never user, Weighted N= 12,825,819; n=5,926 (%)
ICS/LABA^a-b^				
No	92.9	84.2	91.2	87.1
Yes	7.1	15.8	8.8	12.9
SABA^ e^				
No	78.1	63.2	62.9	68.9
Yes	21.9	36.8	37.1	31.1
LABA^ b^				
No	98.8	93.7	95.4	94.4
Yes	1.2	6.3	4.6	5.6
SAMA/SABA^ e-f^				
No	99.5	99.2	99.9	99.1
Yes	0.5	0.8	0.2	0.9
SAMA^ e^				
No	99.4	99.4	97.8	99.9
Yes	0.6	0.6	2.2	0.08
ICS^ a^				
No	94.6	91.5	95.2	93.2
Yes	5.4	8.5	4.8	6.9
LTRA^ c^				
No	97.5	90.6	96.3	90.3
Yes	2.5	9.4	3.7	9.7
OCS^ d^				
No	99.7	98.0	98.0	98.1
Yes	0.3	2.0	2.0	1.9

Multivariable regression models

In Table 4, multivariable regression models with never smokers as the comparison group were conducted. It was determined that there was no statistically significant association between asthma exacerbations with exclusive e-cigarette use (OR: 0.88; 95% CI: 0.56-1.38), exclusive cigarette use (OR: 0.80; 95% CI: 0.60-1.05), or dual-use (OR: 0.98; 95% CI: 0.68-1.41) after adjusting for confounding variables. Similarly, there was no statistically significant association comparing asthma symptoms in the past 30 days with exclusive e-cigarette use (OR: 0.98; 95% CI: 0.58-1.64), exclusive cigarette use (OR: 0.89; 95% CI: 0.65-1.20), or dual-use (OR: 1.04; 95% CI: 0.74-1.47).

**Table 4:** Multivariable regression based on smoking status with ever asthma attack and with asthma symptoms Adjusted for all confounders listed in Table 1.

Ever Asthma Attack	Odds ratio (95 % CI)
Entire cohort (N=25,061,891)	
Exclusive e-cigarette user (N=1,838,585)	0.88 (0.56-1.38)
Exclusive cigarette user (N= 6,706,556)	0.80 (0.60-1.05)
Dual user (N=3,690,931)	0.98 (0.68-1.41)

Asthma Symptoms past 30 days	Odds ratio (95 % CI)
Entire cohort (N=25,061,891)	
Exclusive e-cigarette user (N=1,838,585)	0.98 (0.58-1.64)
Exclusive cigarette user (N= 6,706,556)	0.89 (0.65-1.20)
Dual user (N=3,690,931)	1.04 (0.74-1.47)

## Discussion

In this cross-sectional study, a nationally representative sample of the US population from the 2018 ABCS portion of the BRFSS was analyzed to describe demographics, asthma-related healthcare use, and asthma medication use in exclusive e-cigarette, exclusive cigarette, dual users, and never smokers aged 18 years or older. E-cigarette use was more prevalent among men, White people, with incomes less than $75,000, and employed individuals. Notably, e-cigarette use was the highest among young adults aged 18-24 years, which is consistent with previous studies. As per the CDC’s 2018 National Health Interview Survey [2], younger adults showed higher rates of e-cigarette use in comparison to older adults. This signifies that public health efforts should be focused on addressing reducing rates of e-cigarette use in younger adults. It is particularly important for potential short- and long-term health effects to be investigated given the uncertainty of its consequences and the younger age group of those at risk.

The multivariable regression models showed that there was no association between exclusive e-cigarette use and asthma exacerbations or asthma symptoms in the past 30 days in this representative cohort of adults with asthma. This finding differs from prior studies. The 2015-2018 Canadian Community Health Survey found that e-cigarette users with a diagnosis of asthma had twice the odds of having an asthma attack and that dual use was associated with higher health service use [19]. Another study that conducted a literature search and meta-analysis of epidemiological studies of the general population investigated the association of e-cigarette use with asthma and COPD. They determined that e-cigarette use was associated with respiratory harm and susceptibility to illness [20]. An additional study analyzing data from 2016 to 2018 BRFSS found that, compared to individuals who never smoked e-cigarettes, individuals who do smoke e-cigarettes had increased odds of self-reported diagnosis of asthma (OR: 1.26; 95% CI: 1.25-1.27) [21]. It should be noted that our study focused solely on the BRFSS subset of ACBS, which only included individuals diagnosed with asthma. However, this does support that e-cigarette use leads to adverse respiratory effects that impact biological processes involved in asthma.

It is hypothesized that individuals with asthma who use e-cigarettes have milder disease, which would be reflected by the type of therapy that they receive to manage their respiratory condition. Analysis of the distribution of medication use, as seen in Table 3, revealed that exclusive e-cigarette users were more likely to be taking SABAs (21.9%) in comparison to any other medication. In 1995, the Global Initiative for Asthma Guidelines (GINA) was first published, which described treatment initiatives to control and treat asthma symptoms. Since this publication, SABAs have been recommended as the first-line treatment for patients with mild asthma [22]. GINA has also supported the use of combination therapy of high-dose inhaled corticosteroid (ICS) and long-acting beta agonist (LABA) as a combination therapy for severe asthma since the late 2000s. It is well documented that patients with severe persistent asthma may need additional therapy, which includes OCS, for both maintenance and treatment [23]. Notably, only 7.1% of exclusive e-cigarette users were taking an ICS/LABA combination, and 0.3% were on an OCS. This supports the theory that this cohort of individuals with asthma have a milder form of this respiratory condition as most participants on medication were not using chronic steroids or a LABA/ICS combination.

In addition, only 6% of exclusive e-cigarette asthmatic users had a history of respiratory disease, which is defined as having been diagnosed with COPD, emphysema, or chronic bronchitis. In comparison, there were 29.1%, 32.9%, and 11.1% of exclusive cigarette users, dual users, and never users, respectively, who had a history of respiratory disease. Most exclusive e-cigarette users were also previously noted to be between 18 and 24 years old. This suggests that e-cigarette users with asthma were more likely to have a clinically milder disease that was not complicated by other respiratory pathologies. This could be attributed to the fact that they were significantly younger than the typical age onset of additional chronic conditions. Thus, overall, individuals with mild asthma would have a decreased susceptibility to both asthma attacks and symptoms.

Strengths

There were several strengths of this study. An example would be the utilization of a nationally representative survey, allowing it to be generalizable to the US adult population with asthma over the age of 18. The BRFSS/ACBS also contained a large sample size. It provided robust information about each study participant, which was employed to generate descriptive statistics and control for confounding variables in the regression models. By utilizing never smokers as the reference group, the confounding variable of combustible cigarette use and dual use when analyzing the association between e-cigarettes and asthma exacerbations or asthma symptoms was removed.

Limitations

This study was not without limitations. The BRFSS/ACBS is a cross-sectional study that is subject to recall bias. As a result, participants may not have responded accurately when asked to reflect on their activities, such as any asthma symptoms in the past 30 days, or if they had been accurately and properly diagnosed with asthma as it is based on self-report. In addition, the baseline severity of asthma cannot be determined. Individuals who use e-cigarettes or cigarettes might have asthma that is more or less severe compared to those who do not use these products. Moreover, the number of medications each participant took, and the quantification of their prescribed dosage cannot be deduced. Their medication type, frequency of use, and adherence would all influence susceptibility to asthma attacks, as well as any asthma symptoms in the past 30 days. A final limitation is the inability to establish a temporal relationship between the diagnosis of asthma and the start of e-cigarette or cigarette use.

## Conclusions

There was no association among the three categories of exclusive e-cigarette users, exclusive cigarette users, and dual users with asthma exacerbations or asthma symptoms in the past 30 days in US adults aged 18+. It is possible that the lack of association can be attributed to this study’s composition of participants who had a milder form of asthma as evidenced by the distribution of medication use. This study noted that e-cigarette use was the highest among adults aged 18-24, which is consistent with previous national studies that showed greater use among young adults in comparison to older adults. The results overall emphasize a need to continue e-cigarette use research to assess future health effects further and formulate appropriate public health recommendations, particularly focusing on young adults.

## Appendices

**Table 5:** 2018 Behavioral Risk Surveillance System Questionnaire

Question No.	Question Text	Response Options
LL01	Is this [PHONE NUMBER]?	1. Yes, 2. No
LL02	Is this a private residence?	1. Yes, 2. No, 3. No, this is a business
LL03	Do you live in college housing?	1. Yes, 2. No
LL04	Do you currently live in __(state)____?	1. Yes, 2. No
LL05	Is this a cell phone?	1. Yes, it is a cell phone, 2. Not a cell phone
LL06	Are you 18 years of age or older?	1. Yes, male respondent, 2. Yes, female respondent, 3. No
LL07	I need to randomly select one adult who lives in your household to be interviewed. Excluding adults living away from home, such as students away at college, how many members of your household, including yourself, are 18 years of age or older?	1. (Number of adults)
LL08	How many of these adults are men?	1. Number, 77. Don’t know/Not sure, 99. Refused.
LL09	So the number of women in the household is [X]. Is that correct?	1. Do not read: Confirm the number of adult women or clarify the total number of adults in the household, 2. Read: The persons in your household that I need to speak with is [XXX].
CP01	Is this a safe time to talk with you?	1. Yes, 2. No (set appointment if possible), TERMINATE. Thank you very much. We will call you back at a more convenient time.
CP02	Is this [PHONE NUMBER]?	1. Yes, 2. No, TERMINATE.
CP03	Is this a cell phone?	1. Yes, 2. No, TERMINATE. Read: Thank you very much but we are only interviewing persons aged 18 or older at this time.
CP04	Are you 18 years of age or older?	1. Yes, male respondent, 2. Yes, female respondent, 3. No, TERMINATE. Read: Thank you very much but we are only interviewing persons aged 18 or older at this time.
CP05	Do you live in a private residence?	1. Yes, 2. No, TERMINATE. Thank you very much, but we are only interviewing persons who live in private residences or college housing at this time.
CP06	Do you live in college housing?	1. Yes, 2. No, TERMINATE.
CP07	Do you currently live in [state]?	1. Yes, 2. No.
CP08	In what state do you currently live?	1. Alabama, 2. Alaska, 4. Arizona, 5. Arkansas, 6. California, 8. Colorado, 9. Connecticut, 10. Delaware, 11. District of Columbia, 12. Florida, 13. Georgia, 15. Hawaii, 16. Idaho, 17. Illinois, 18. Indiana, 19. Iowa, 20. Kansas, 21. Kentucky, 22. Louisiana, 23. Maine, 24. Maryland, 25. Massachusetts, 26. Michigan, 27. Minnesota, 28. Mississippi, 29. Missouri, 30. Montana, 31. Nebraska, 32. Nevada, 33. New .Hampshire, 34. New Jersey, .35. New Mexico, 36. New York, 37. North Carolina, 38. North Dakota, 39. Ohio, 40. Oklahoma, 41. Oregon, 42. Pennsylvania, 44. Rhode Island, 45. South Carolina, 46. South Dakota, 47. Tennessee, 48. Texas, 49. Utah, 50. Vermont, 51. Virginia, 53. Washington, 54. West Virginia, 55. Wisconsin, 56. Wyoming, 66. Guam, 72. Puerto Rico, 78. Virgin Islands, 99. Refused.
CP09	Do you also have a landline telephone in your home that is used to make and receive calls?	1. Yes, 2. No, 7. Don’t know/Not sure, 9. Refused.
CP10	How many members of your household, including yourself, are 18 years of age or older?	1. Number, 77. Don’t know/Not sure, 99. Refused.
C01.01	Would you say that in general your health is—	1. Excellent, 2. Very Good, 3. Good, 4. Fair, 5. Poor, 7. Don’t know/Not sure, 9. Refused.
C02.01	Now thinking about your physical health, which includes physical illness and injury, for how many days during the past 30 days was your physical health not good?	1. Number of days (01-30), 88. None, 77. Don’t know/not sure, 99. Refused.
C02.02	Now thinking about your mental health, which includes stress, depression, and problems with emotions, for how many days during the past 30 days was your mental health not good?	1. Number of days (01-30), 88. None, 77. Don’t know/not sure, 99. Refused.
C02.03	During the past 30 days, for about how many days did poor physical or mental health keep you from doing your usual activities, such as selfcare, work, or recreation?	1. Number of days (01-30), 88. None, 77. Don’t know/not sure, 99. Refused.
C03.01	Do you have any kind of health care coverage, including health insurance, prepaid plans such as HMOs, or government plans such as Medicare, or Indian Health Service?	1. Yes, 2. No, 7. Don’t know/Not sure, 9. Refused.
C03.02	Do you have one person you think of as your personal doctor or health care provider?	1. Yes, only one, 2. More than one, 3. No, 7. Don’t know / Not sure, 9. Refused.
C03.03	Was there a time in the past 12 months when you needed to see a doctor but could not because of cost?	1. Yes, 2. No, 7. Don’t know / Not sure, 9. Refused.
C03.04	About how long has it been since you last visited a doctor for a routine checkup?	1. Within the past year, 2. Within the past 2 years, 3. Within the past 5 years, 4. 5 or more years ago, 7. Don’t know / Not sure, 8. Never, 9. Refused.
C04.01	During the past month, other than your regular job, did you participate in any physical activities or exercises such as running, calisthenics, golf, gardening, or walking for exercise?	1. Yes, 2. No, 7. Don’t know / Not sure, 9. Refused.
C05.01	On average, how many hours of sleep do you get in a 24-hour period?	1. Number of hours (01-24), 77. Don’t know / Not sure, 99. Refused.
C06.01	Has a doctor, nurse, or other health professional ever told you that you had any of the following? Ever told you had a heart attack also called a myocardial infarction?	1. Yes, 2. No, 7. Don’t know / Not sure, 9. Refused.
C06.02	(Ever told) you had angina or coronary heart disease?	1. Yes, 2. No, 7. Don’t know / Not sure, 9. Refused.
C06.03	(Ever told) you had a stroke?	1. Yes, 2. No, 7. Don’t know / Not sure, 9. Refused.
C06.04	(Ever told) you had asthma?	1. Yes, 2. No, 7. Don’t know / Not sure, 9. Refused.
C06.05	Do you still have asthma?	1. Yes, 2. No, 7. Don’t know / Not sure, 9. Refused.
C06.06	(Ever told) you had skin cancer?	1. Yes, 2. No, 7. Don’t know / Not sure, 9. Refused.
C06.07	(Ever told) you had any other types of cancer?	1. Yes, 2. No, 7. Don’t know / Not sure, 9. Refused.
C06.08	(Ever told) you have chronic obstructive pulmonary disease, C.O.P.D., emphysema or chronic bronchitis?	1. Yes, 2. No, 7. Don’t know / Not sure, 9. Refused.
C06.09	(Ever told) you have some form of arthritis, rheumatoid arthritis, gout, lupus, or fibromyalgia?	1. Yes, 2. No, 7. Don’t know / Not sure, 9. Refused.
C06.10	(Ever told) you have a depressive disorder (including depression, major depression, dysthymia, or minor depression)?	1. Yes, 2. No, 7. Don’t know / Not sure, 9. Refused.
C06.11	Not including kidney stones, bladder infection or incontinence, were you ever told you have kidney disease?	1. Yes, 2. No, 7. Don’t know / Not sure, 9. Refused.
C06.12	(Ever told) you have diabetes?	1. Yes, 2. Yes, but female told only during pregnancy, 3. No, 4. No, prediabetes or borderline diabetes, 7. Don’t know / Not sure, 9. Refused.
C06.13	How old were you when you were told you have diabetes?	_ _ Code age in years [97 = 97 and older], 98. Don‘t know / Not sure, 99. Refused
C07.01	Including all types of dentists, such as orthodontists, oral surgeons, and all other dental specialists, as well as dental hygienists, how long has it been since you last visited a dentist or a dental clinic for any reason?	1. Within the past year (anytime less than 12 months ago), 2. Within the past 2 years (1 year but less than 2 years ago), 3. Within the past 5 years (2 years but less than 5 years ago), 4. 5 or more years ago, 7. Don’t know / Not sure, 8. Never, 9. Refused.
C07.02	Not including teeth lost for injury or orthodontics, how many of your permanent teeth have been removed because of tooth decay or gum disease?	1. 1 to 5, 2. 6 or more but not all, 3. All, 8. None, 7. Don’t know / Not sure, 9. Refused.
C08.01	What is your sex? What was your sex at birth? Was it...	1. Male, 2. Female, 7. Don’t know / Not sure, 9. Refused.
C08.02	What is your age?	_ _ Code age in years, 07. Don’t know / Not sure, 09. Refused.
C08.03	Are you Hispanic, Latino/a, or Spanish origin?	1. Mexican, Mexican American, Chicano/a, 2. Puerto Rican, 3. Cuban, 4. Another Hispanic, Latino/a, or Spanish origin, 5. No, 7. Don’t know / Not sure, 9. Refused.
C08.04	Which one or more of the following would you say is your race?	10. White, 20. Black or African American, 30. American Indian or Alaska Native, 40. Asian, 41. Asian Indian, 42. Chinese, 43. Filipino, 44. Japanese, 45. Korean, 46. Vietnamese, 47. Other Asian, 50. Pacific Islander, 51. Native Hawaiian, 52. Guamanian or Chamorro, 53. Samoan, 54. Other Pacific Islander, 60. Other, 88. No additional choices, 77. Don’t know / Not sure, 99. Refused.
C08.05	Which one of these groups would you say best represents your race?	10. White, 20. Black or African American, 30. American Indian or Alaska Native, 40. Asian, 41. Asian Indian, 42. Chinese, 43. Filipino, 44. Japanese, 45. Korean, 46. Vietnamese, 47. Other Asian, 50. Pacific Islander, 51. Native Hawaiian, 52. Guamanian or Chamorro, 53. Samoan, 54. Other Pacific Islander, 60. Other, 77. Don’t know / Not sure, 99. Refused.
C08.06	Are you...	1. Married, 2. Divorced, 3. Widowed, 4. Separated, 5. Never married, 6. A member of an unmarried couple, 9. Refused.
C08.07	What is the highest grade or year of school you completed?	1. Never attended school or only attended kindergarten, 2. Grades 1 through 8 (Elementary), 3. Grades 9 through 11 (Some high school), 4. Grade 12 or GED (High school graduate), 5. College 1 year to 3 years (Some college or technical school), 6. College 4 years or more (College graduate), 9. Refused.
C08.08	Do you own or rent your home?	1. Own, 2. Rent, 3. Other arrangement, 7. Don’t know / Not sure, 9. Refused.
C08.09	In what county do you currently live?	_ _ _ ANSI County Code, 777. Don’t know / Not sure, 999. Refused.
C08.10	What is the ZIP Code where you currently live?	_ _ _ _ _, 77777. Do not know, 99999. Refused.
C08.11	Not including cell phones or numbers used for computers, fax machines, or security systems, do you have more than one telephone number in your household?	1. Yes, 2. No, 7. Don’t know / Not sure, 9. Refused.
C08.12	How many of these telephone numbers are residential numbers?	Enter number (1-5), 6. Six or more, 7. Don’t know / Not sure, 8. None, 9. Refused.
C08.13	How many cell phones do you have for personal use?	Enter number (1-5), 6. Six or more, 7. Don’t know / Not sure, 8. None, 9. Refused.
C08.14	Have you ever served on active duty in the United States Armed Forces, either in the regular military or in a National Guard or military reserve unit?	1. Yes, 2. No, 7. Don’t know / Not sure, 9. Refused.
C08.15	Are you currently...	1. Employed for wages, 2. Self-employed, 3. Out of work for 1 year or more, 4. Out of work for less than 1 year, 5. Homemaker, 6. Student, 7. Retired, 8. Unable to work, 9. Refused.
C08.16	How many children less than 18 years of age live in your household?	Enter number (1-18), 88. None, 99. Refused.
C08.17	Is your annual household income from all sources—	01. Less than $10,000, 02. Less than $15,000, 03. Less than $20,000, 04. Less than $25,000, 05. Less than $35,000, 06. Less than $50,000, 07. Less than $75,000, 08. $75,000 or more, 77. Don’t know / Not sure, 99. Refused.
C08.18	About how much do you weigh without shoes?	Enter weight (lbs/kgs), 7777. Don’t know / Not sure, 9999. Refused.
C08.19	About how tall are you without shoes?	Enter height (ft/inches or cm), 77/77. Don’t know / Not sure, 99/99. Refused.
C08.20	To your knowledge, are you now pregnant?	1. Yes, 2. No, 7. Don’t know / Not sure, 9. Refused.
C08.21	Are you deaf or do you have serious difficulty hearing?	1. Yes, 2. No, 7. Don’t know / Not sure, 9. Refused.
C08.22	Are you blind or do you have serious difficulty seeing, even when wearing glasses?	1. Yes, 2. No, 7. Don’t know / Not sure, 9. Refused.
C08.23	Because of a physical, mental, or emotional condition, do you have serious difficulty concentrating, remembering, or making decisions?	1. Yes, 2. No, 7. Don’t know / Not sure, 9. Refused.
C08.24	Do you have serious difficulty walking or climbing stairs?	1. Yes, 2. No, 7. Don’t know / Not sure, 9. Refused.
C08.25	Do you have difficulty dressing or bathing?	1. Yes, 2. No, 7. Don’t know / Not sure, 9. Refused.
C08.26	Because of a physical, mental, or emotional condition, do you have difficulty doing errands alone such as visiting a doctor’s office or shopping?	1. Yes, 2. No, 7.
C09.01	Have you smoked at least 100 cigarettes in your entire life?	1. Yes, 2. No, 7. Don’t know / Not Sure, 9. Refused.
C09.02	Do you now smoke cigarettes every day, some days, or not at all?	1. Every day, 2. Some days, 3. Not at all, 7. Don’t know / Not sure, 9. Refused.
C09.03	During the past 12 months, have you stopped smoking for one day or longer because you were trying to quit smoking?	1. Yes, 2. No, 7. Don’t know / Not sure, 9. Refused.
C09.04	How long has it been since you last smoked a cigarette, even one or two puffs?	1. Within the past month (less than 1 month ago), 2. Within the past 3 months (1 month but less than 3 months ago), 3. Within the past 6 months (3 months but less than 6 months ago), 4. Within the past year (6 months but less than 1 year ago), 5. Within the past 5 years (1 year but less than 5 years ago), 6. Within the past 10 years (5 years but less than 10 years ago), 7. 10 years or more, 8. Never smoked regularly, 77. Don’t know / Not sure, 99. Refused.
C09.05	Do you currently use chewing tobacco, snuff, or snus every day, some days, or not at all?	1. Every day, 2. Some days, 3. Not at all, 7. Don’t know / Not sure, 9. Refused.
C10.01	During the past 30 days, how many days per week or per month did you have at least one drink of any alcoholic beverage such as beer, wine, a malt beverage or liquor?	1. Days per week, 2. Days in past 30 days, 888. No drinks in past 30 days, 777. Don’t know / Not sure, 999. Refused.
C10.02	During the past 30 days, on the days when you drank, about how many drinks did you drink on the average?	1. Number of drinks, 88. None, 77. Don’t know / Not sure, 99. Refused.
C10.03	Considering all types of alcoholic beverages, how many times during the past 30 days did you have X [CATI X = 5 for men, X = 4 for women] or more drinks on an occasion?	1. Number of times, 77. Don’t know / Not sure, 99. Refused.
C10.04	During the past 30 days, what is the largest number of drinks you had on any occasion?	1. Number of drinks, 77. Don’t know / Not sure, 99. Refused.
C11.01	During the past 12 months, have you had either a flu shot or a flu vaccine that was sprayed in your nose?	1. Yes, 2. No, 7. Don’t know / Not sure, 9. Refused
C11.02	During what month and year did you receive your most recent flu shot injected into your arm or flu vaccine that was sprayed in your nose?	Month / Year, 77 / 7777 Don’t know / Not sure, 09 / 9999 Refused
C11.03	At what kind of place did you get your last flu shot or vaccine?	01. A doctor’s office or health maintenance organization (HMO), 02. A health department, 03. Another type of clinic or health center (a community health center), 04. A senior, recreation, or community center, 05. A store (supermarket, drug store), 06. A hospital (inpatient), 07. An emergency room, 08. Workplace, 09. Some other kind of place, 10. Received vaccination in Canada/Mexico, 77. Don’t know / Not sure, 99. Refused
C11.04	Have you ever had a pneumonia shot also known as a pneumococcal vaccine?	1. Yes, 2. No, 7. Don’t know / Not sure, 9. Refused
C12.01	In the past 12 months, how many times have you fallen?	_ _ Number of times, 88 None, 77 Don’t know / Not sure, 99 Refused
C12.02	Did this fall cause an injury that limited your regular activities for at least a day or caused you to go to see a doctor?	_ _ Number of falls [76 = 76 or more], 88 None, 77 Don’t know / Not sure, 99 Refused
C13.01	How often do you use seat belts when you drive or ride in a car? Would you say—	1. Always, 2. Nearly always, 3. Sometimes, 4. Seldom, 5. Never, 7. Don’t know / Not sure, 8. Never drive or ride in a car, 9. Refused
C13.02	During the past 30 days, how many times have you driven when you’ve had perhaps too much to drink?	_ _ Number of times, 88 None, 77 Don’t know / Not sure, 99 Refused
C14.01	Have you ever had a mammogram?	1. Yes, 2. No, 7. Don’t know/ not sure, 9. Refused
C14.02	How long has it been since you had your last mammogram?	1. Within the past year (anytime less than 12 months ago), 2. Within the past 2 years (1 year but less than 2 years ago), 3. Within the past 3 years (2 years but less than 3 years ago), 4. Within the past 5 years (3 years but less than 5 years ago), 5. 5 or more years ago, 7. Don’t know / Not sure, 9. Refused
C14.03	Have you ever had a Pap test?	1. Yes, 2. No, 7. Don’t know / Not sure, 9. Refused
C14.04	How long has it been since you had your last Pap test?	1. Within the past year (anytime less than 12 months ago), 2. Within the past 2 years (1 year but less than 2 years ago), 3. Within the past 3 years (2 years but less than 3 years ago), 4. Within the past 5 years (3 years but less than 5 years ago), 5. 5 or more years ago, 7. Don’t know / Not sure, 9. Refused
C14.05	Have you ever had an H.P.V. test?	1. Yes, 2. No, 7. Don’t know / Not sure, 9. Refused
C14.06	How long has it been since you had your last H.P.V. test?	1. Within the past year (anytime less than 12 months ago), 2. Within the past 2 years (1 year but less than 2 years ago), 3. Within the past 3 years (2 years but less than 3 years ago), 4. Within the past 5 years (3 years but less than 5 years ago), 5. 5 or more years ago, 7. Don’t know / Not sure, 9. Refused
C14.07	Have you had a hysterectomy?	1. Yes, 2. No, 7. Don’t know / Not sure, 9. Refused
C15.01	Has a doctor, nurse, or other health professional ever talked with you about the advantages of the Prostate-Specific Antigen or P.S.A. test?	1. Yes, 2. No, 7. Don’t know/ not sure, 9. Refused
C15.02	Has a doctor, nurse, or other health professional ever talked with you about the disadvantages of the P.S.A. test?	1. Yes, 2. No, 7. Don’t know/ not sure, 9. Refused
C15.03	Has a doctor, nurse, or other health professional ever recommended that you have a P.S.A. test?	1. Yes, 2. No, 7. Don’t know / Not sure, 9. Refused
C15.04	Have you ever had a P.S.A. test?	1. Yes, 2. No, 7. Don’t know / Not sure, 9. Refused
C15.05	How long has it been since you had your last P.S.A. test?	1. Within the past year (anytime less than 12 months ago), 2. Within the past 2 years (1 year but less than 2 years ago), 3. Within the past 3 years (2 years but less than 3 years ago), 4. Within the past 5 years (3 years but less than 5 years ago), 5. 5 or more years ago, 7. Don’t know / Not sure, 9. Refused
C15.06	What was the main reason you had this P.S.A. test – was it …?	1. Part of a routine exam, 2. Because of a prostate problem, 3. Because of a family history of prostate cancer, 4. Because you were told you had prostate cancer, 5. Some other reason, 7. Don’t know / Not sure, 9. Refused
C16.01	A blood stool test is a test that may use a special kit at home to determine whether the stool contains blood. Have you ever had this test using a home kit?	1. Yes, 2. No, 7. Don’t know/ not sure, 9. Refused
C16.02	How long has it been since you had your last blood stool test using a home kit?	1. Within the past year (anytime less than 12 months ago), 2. Within the past 2 years (1 year but less than 2 years ago), 3. Within the past 3 years (2 years but less than 3 years ago), 4. Within the past 5 years (3 years but less than 5 years ago), 5. 5 or more years ago, 7. Don’t know / Not sure, 9. Refused
C16.03	Sigmoidoscopy and colonoscopy are exams in which a tube is inserted in the rectum to view the colon for signs of cancer or other health problems. Have you ever had either of these exams?	1. Yes, 2. No, 7. Don’t know / Not sure, 9. Refused
C16.04	For a sigmoidoscopy, a flexible tube is inserted into the rectum to look for problems. A colonoscopy is similar, but uses a longer tube. Was your most recent exam a sigmoidoscopy or a colonoscopy?	1. Sigmoidoscopy, 2. Colonoscopy, 7. Don’t know / Not sure, 9. Refused
C16.05	How long has it been since you had your last sigmoidoscopy or colonoscopy?	1. Within the past year (anytime less than 12 months ago), 2. Within the past 2 years (1 year but less than 2 years ago), 3. Within the past 3 years (2 years but less than 3 years ago), 4. Within the past 5 years (3 years but less than 5 years ago), 5. Within the past 10 years (5 years but less than 10 years ago), 6. 10 or more years ago, 7. Don't know / Not sure, 9. Refused
C17.01	Have you ever been tested for H.I.V.? Do not count tests you may have had as part of a blood donation.	1. Yes, 2. No, 7. Don’t know/ not sure, 9. Refused
C17.02	Not including blood donations, in what month and year was your last H.I.V. test?	_ _ /_ _ _ _ Code month and year, 77/ 7777 Don’t know / Not sure, 99/ 9999 Refused
C17.03	Do any of the following situations apply to you: Have you injected any drug other than those prescribed for you in the past year, been treated for an STD, given or received money or drugs in exchange for sex, had anal sex without a condom, had four or more sex partners in the past year?	1. Yes, 2. No, 7. Don’t know / Not sure, 9. Refused
M01.01	Have you had a test for high blood sugar or diabetes within the past three years?	1. Yes, 2. No, 7. Don’t know/ not sure, 9. Refused
M01.02	Have you ever been told by a doctor or other health professional that you have pre-diabetes or borderline diabetes?	1. Yes, 2. Yes, during pregnancy, 3. No, 7. Don’t know / Not sure, 9. Refused
M02.01	Are you now taking insulin?	1. Yes, 2. No, 7. Don’t know/ not sure, 9. Refused
M02.02	About how often do you check your blood for glucose or sugar?	_ _ Times per day, _ _ Times per week, _ _ Times per month, _ _ Times per year, 888 Never, 777 Don’t know / Not sure, 999 Refused
M02.03	Including times when checked by a family member or friend, about how often do you check your feet for any sores or irritations?	_ _ Times per day, _ _ Times per week, _ _ Times per month, _ _ Times per year, 555 No feet, 888 Never, 777 Don’t know / Not sure, 999 Refused
M02.04	About how many times in the past 12 months have you seen a doctor, nurse, or other health professional for your diabetes?	_ _ Number of times [76 = 76 or more], 88 None, 77 Don’t know / Not sure, 99 Refused
M02.05	About how many times in the past 12 months has a doctor, nurse, or other health professional checked you for A-one-C?	_ _ Number of times [76 = 76 or more], 88 None, 98 Never heard of A-one-C test, 77 Don’t know / Not sure, 99 Refused
M02.06	About how many times in the past 12 months has a health professional checked your feet for any sores or irritations?	_ _ Number of times [76 = 76 or more], 88 None, 77 Don’t know / Not sure, 99 Refused
M02.07	When was the last time you had an eye exam in which the pupils were dilated, making you temporarily sensitive to bright light?	1. Within the past month (anytime less than 1 month ago), 2. Within the past year (1 month but less than 12 months ago), 3. Within the past 2 years (1 year but less than 2 years ago), 4. 2 or more years ago, 7 Don’t know / Not sure, 8 Never, 9 Refused
M02.08	Has a doctor ever told you that diabetes has affected your eyes or that you had retinopathy?	1. Yes, 2. No, 7 Don’t know/ not sure, 9 Refused
M02.09	Have you ever taken a course or class in how to manage your diabetes yourself?	1. Yes, 2. No, 7 Don’t know/ not sure, 9 Refused
M03.01	Do you have Medicare?	1. Yes, 2. No, 7. Don’t know/ not sure, 9. Refused
M03.02	What is the primary source of your health care coverage? Is it…	01. A plan purchased through an employer or union (including plans purchased through another person's employer), 02. A plan that you or another family member buys on your own, 03. Medicare, 04. Medicaid or other state program, 05. TRICARE (formerly CHAMPUS), VA, or Military, 06. Alaska Native, Indian Health Service, Tribal Health Services, 07. Some other source, 08. None (no coverage), 77. Don't know/Not sure, 99. Refused
M03.03	Other than cost, have you delayed getting medical care for one of the following reasons in the past 12 months? Was it because…..	1. You couldn’t get through on the telephone, 2. You couldn’t get an appointment soon enough, 3. Once you got there, you had to wait too long to see the doctor, 4. The clinic or doctor’s office wasn’t open when you got there, 5. You didn’t have transportation, 8. No, I did not delay getting medical care/did not need medical care, 7. Don’t know/Not sure, 9. Refused, 6. Other (specify)
M03.04	In the past 12 months was there any time when you did not have any health insurance or coverage?	1. Yes, 2. No, 7. Don’t know/ not sure, 9. Refused
M03.04a	About how long has it been since you last had health care coverage?	1. 6 months or less, 2. More than 6 months, but not more than 1 year ago, 3. More than 1 year, but not more than 3 years ago, 4. More than 3 years, 5. Never, 7. Don’t know/Not sure, 9. Refused
M03.05	How many times have you been to a doctor, nurse, or other health professional in the past 12 months?	_ _ Number of times [76 = 76 or more], 88. None, 77. Don’t know / Not sure, 99. Refused
M03.06	Not including over the counter (OTC) medications, was there a time in the past 12 months when you did not take your medication as prescribed because of cost?	1. Yes, 2. No, 3. No medication was prescribed, 7. Don’t know/ not sure, 9. Refused
M03.07	In general, how satisfied are you with the health care you received? Would you say—	1. Very satisfied, 2. Somewhat satisfied, 3. Not at all satisfied, 8. Not applicable, 7. Don’t know/Not sure, 9. Refused
M03.08	Do you currently have any health care bills that are being paid off over time?	1. Yes, 2. No, 7. Don’t know/ not sure, 9. Refused
M04.01	During the past 12 months, have you experienced confusion or memory loss that is happening more often or is getting worse?	1. Yes, 2. No, 7. Don’t know/ not sure, 9. Refused
M04.02	During the past 12 months, as a result of confusion or memory loss, how often have you given up day-to-day household activities or chores you used to do, such as cooking, cleaning, taking medications, driving, or paying bills? Would you say it is…	1. Always, 2. Usually, 3. Sometimes, 4. Rarely, 5. Never, 7. Don’t know/Not sure, 9. Refused
M04.03	As a result of confusion or memory loss, how often do you need assistance with these day-to-day activities? Would you say it is…	1. Always, 2. Usually, 3. Sometimes, 4. Rarely, 5. Never, 7. Don’t know/Not sure, 9. Refused
M04.04	When you need help with these day-to-day activities, how often are you able to get the help that you need? Would you say it is…	1. Always, 2. Usually, 3. Sometimes, 4. Rarely, 5. Never, 7. Don’t know/Not sure, 9. Refused
M04.05	During the past 12 months, how often has confusion or memory loss interfered with your ability to work, volunteer, or engage in social activities outside the home? Would you say it is…	1. Always, 2. Usually, 3. Sometimes, 4. Rarely, 5. Never, 7. Don’t know/Not sure, 9. Refused
M04.06	Have you or anyone else discussed your confusion or memory loss with a health care professional?	1. Yes, 2. No, 7. Don’t know/ not sure, 9. Refused
M05.01	During the past 30 days, did you provide regular care or assistance to a friend or family member who has a health problem or disability?	1. Yes, 2. No, 7. Don’t know/Not sure, 8. Caregiving recipient died in past 30 days, 9. Refused
M05.02	What is his or her relationship to you?	01. Mother, 02. Father, 03. Mother-in-law, 04. Father-in-law, 05. Child, 06. Husband, 07. Wife, 08. Live-in partner, 09. Brother or brother-in-law, 10. Sister or sister-in-law, 11. Grandmother, 12. Grandfather, 13. Grandchild, 14. Other relative, 15. Non-relative/ Family friend, 77. Don’t know/Not sure, 99. Refused
M05.03	For how long have you provided care for that person? Would you say…	1. Less than 30 days, 2. 1 month to less than 6 months, 3. 6 months to less than 2 years, 4. 2 years to less than 5 years, 5. More than 5 years, 7. Don’t know/Not sure, 9. Refused
M05.04	In an average week, how many hours do you provide care or assistance? Would you say…	1. Up to 8 hours per week, 2. 9 to 19 hours per week, 3. 20 to 39 hours per week, 4. 40 hours or more, 7. Don’t know/Not sure, 9. Refused
M05.05	What is the main health problem, long-term illness, or disability that the person you care for has?	01. Arthritis/rheumatism, 02. Asthma, 03. Cancer, 04. Chronic respiratory conditions such as emphysema or COPD, 05. Alzheimer’s disease, dementia or other cognitive impairment disorder, 06. Developmental disabilities such as autism, Down’s Syndrome, and spina bifida, 07. Diabetes, 08. Heart disease, hypertension, stroke, 09. Human Immunodeficiency Virus Infection (H.I.V.), 10. Mental illnesses, such as anxiety, depression, or schizophrenia, 11. Other organ failure or diseases such as kidney or liver problems, 12. Substance abuse or addiction disorders, 13. Injuries, including broken bones, 14. Old age/infirmity/frailty, 15. Other, 77. Don’t know/Not sure, 99. Refused
M05.06	In the past 30 days, did you provide care for this person by managing personal care such as giving medications, feeding, dressing, or bathing?	1. Yes, 2. No, 7. Don’t know/ not sure, 9. Refused
M05.07	In the past 30 days, did you provide care for this person by managing household tasks such as cleaning, managing money, or preparing meals?	
M05.08	Of the following support services, which one do you, as a caregiver, most need that you are not currently getting?	1. Classes about giving care, such as giving medications, 2. Help in getting access to services, 3. Support groups, 4. Individual counseling to help cope with giving care, 5. Respite care, 6. You don’t need any of these support services, 7. Don’t know/Not sure, 9. Refused
M05.09	In the next 2 years, do you expect to provide care or assistance to a friend or family member who has a health problem or disability?	1. Yes, 2. No, 7. Don’t know/ not sure, 9. Refused
M06.01	Have you ever used an e-cigarette or other electronic vaping product, even just one time, in your entire life?	1. Yes, 2. No, 7. Don’t know/Not sure, 9. Refused
M06.02	Do you now use e-cigarettes or other electronic vaping products every day, some days, or not at all?	1. Every day, 2. Some days, 3. Not at all, 7. Don’t know/Not sure, 9. Refused
M07.01	During the past 30 days, on how many days did you use marijuana or cannabis?	_ _ (01-30 Number of days), 88. None, 77. Don’t know/Not sure, 99. Refused
M07.02	During the past 30 days, which one of the following ways did you use marijuana the most often? Did you usually…	1. Smoke it, 2. Eat it, 3. Drink it, 4. Vaporize it, 5. Dab it, 6. Use it some other way, 7. Don’t know/Not sure, 9. Refused
M07.03	When you used marijuana or cannabis during the past 30 days, was it usually:	1. For medical reasons, 2. For nonmedical reasons, 3. For both medical and nonmedical reasons, 7. Don’t know/Not sure, 9. Refused
M08.01	Over the last 2 weeks, how many days have you had trouble falling asleep or staying asleep or sleeping too much?	_ _ (01-14 Number of days), 88. None, 77. Don’t know/Not sure, 99. Refused
M08.02	Over the last 2 weeks, how many days did you unintentionally fall asleep during the day?	_ _ (01-14 Number of days), 88. None, 77. Don’t know/Not sure, 99. Refused
M08.03	Have you ever been told that you snore loudly?	1. Yes, 2. No, 7. Don’t know/Not sure, 9. Refused
M08.04	Has anyone ever observed that you stop breathing during your sleep?	1. Yes, 2. No, 7. Don’t know/Not sure, 9. Refused
M09.01	Over the last 2 weeks, how often have you been bothered by having little interest or pleasure in doing things? Would you say this happens...	1. Never, 2. For several days, 3. For more than half the days, 4. Nearly every day, 7. Don’t know/Not sure, 9. Refused
M09.02	Over the last 2 weeks, how often have you been bothered by feeling down, depressed or hopeless? Would you say this happens…	1. Never, 2. For several days, 3. For more than half the days, 4. Nearly every day, 7. Don’t know/Not sure, 9. Refused
M09.03	Over the last 2 weeks, how often have you been bothered by feeling nervous, anxious or on edge? Would you say this happens…	1. Never, 2. For several days, 3. For more than half the days, 4. Nearly every day, 7. Don’t know/Not sure, 9. Refused
M09.04	Over the last 2 weeks, how often have you been bothered by not being able to stop or control worrying? Would you say this happens…	1. Never, 2. For several days, 3. For more than half the days, 4. Nearly every day, 7. Don’t know/Not sure, 9. Refused
M10.01	During the past 3 months, did you have a cough on most days?	1. Yes, 2. No, 7. Don’t know/Not sure, 9. Refused
M10.02	During the past 3 months, did you cough up phlegm [FLEM] or mucus on most days?	1. Yes, 2. No, 7. Don’t know/Not sure, 9. Refused
M10.03	Do you have shortness of breath either when hurrying on level ground or when walking up a slight hill or stairs?	1. Yes, 2. No, 7. Don’t know/Not sure, 9. Refused
M10.04	Have you ever been given a breathing test to diagnose breathing problems?	1. Yes, 2. No, 7. Don’t know/Not sure, 9. Refused
M10.05	Over your lifetime, how many years have you smoked tobacco products?	_ _ (01-76 Number of years), 88. Never smoked or smoked less than one year, 77. Don’t know/Not sure, 99. Refused
M11.01	Not including spray-on tans, during the past 12 months, how many times have you used an indoor tanning device such as a sunlamp, tanning bed, or booth?	_ _ _ (Number 0-365), 777. Don’t know/Not sure, 999. Refused
M12.01	During the past 12 months, how many times have you had a sunburn?	_ _ _ (Number 0-365), 777. Don’t know/Not sure, 999. Refused
M12.02	When you go outside on a warm sunny day for more than one hour, how often do you protect yourself from the sun?	1. Always, 2. Most of the time, 3. Sometimes, 4. Rarely, 5. Never, 6. Don’t stay outside for more than one hour, 8. Don’t go outside at all, 7. Don’t know/Not sure, 9. Refused
M12.03	On weekdays, in the summer, how long are you outside per day between 10am and 4pm?	01. Less than half an hour, 02. More than half an hour up to 1 hour, 03. More than 1 hour up to 2 hours, 04. More than 2 hours up to 3 hours, 05. More than 3 hours up to 4 hours, 06. More than 4 hours up to 5 hours, 07. More than 5 up to 6 hours, 77. Don’t know/Not sure, 99. Refused
M12.04	On weekends in the summer, how long are you outside each day between 10am and 4pm?	01. Less than half an hour, 02. More than half an hour up to 1 hour, 03. More than 1 hour up to 2 hours, 04. More than 2 hours up to 3 hours, 05. More than 3 hours up to 4 hours, 06. More than 4 hours up to 5 hours, 07. More than 5 up to 6 hours, 77. Don’t know/Not sure, 99. Refused
M13.01	You’ve told us that you have smoked in the past or are currently smoking. How old were you when you first started to smoke cigarettes regularly?	_ _ _ (Age in Years 001 – 100), 777. Don't know/Not sure, 999. Refused
M13.02	How old were you when you last smoked cigarettes regularly?	_ _ _ (Age in Years 001 – 100), 777. Don't know/Not sure, 999. Refused
M13.03	On average, when you {smoke/smoked} regularly, about how many cigarettes {do/did} you usually smoke each day?	_ _ _ (Number of cigarettes), 777. Don't know/Not sure, 999. Refused
M13.04	In the last 12 months, did you have a CT or CAT scan?	1. Yes, to check for lung cancer, 2. No (did not have a CT scan), 3. Had a CT scan, but for some other reason, 7. Don't know/not sure, 9. Refused
M14.01	You’ve told us that you have had cancer. How many different types of cancer have you had?	1. Only one, 2. Two, 3. Three or more, 7. Don’t know / Not sure, 9. Refused
M14.02	At what age were you told that you had cancer?	_ _ (Age in Years; 97 = 97 and older), 98. Don't know/Not sure, 99. Refused
M14.03	What type of cancer was it?	01. Breast cancer, 02. Cervical cancer, 03. Endometrial cancer, 04. Ovarian cancer, 05. Head and neck cancer, 06. Oral cancer, 07. Pharyngeal cancer, 08. Thyroid, 09. Larynx, 10. Colon cancer, 11. Esophageal cancer, 12. Liver cancer, 13. Pancreatic cancer, 14. Rectal cancer, 15. Stomach cancer, 16. Hodgkin's Lymphoma, 17. Leukemia, 18. Non-Hodgkin’s Lymphoma, 19. Prostate cancer, 20. Testicular cancer, 21. Melanoma, 22. Other skin cancer, 23. Heart, 24. Lung, 25. Bladder cancer, 26. Renal cancer, 27. Bone cancer, 28. Brain cancer, 29. Neuroblastoma, 30. Other, 77. Don’t know / Not sure, 99. Refused
M14.04	Are you currently receiving treatment for cancer?	1. Yes, 2. No, I’ve completed treatment, 3. No, I’ve refused treatment, 4. No, I haven’t started treatment, 7. Don’t know / Not sure, 9. Refused
M14.05	What type of doctor provides the majority of your health care? Is it a….	01. Cancer Surgeon, 02. Family Practitioner, 03. General Surgeon, 04. Gynecologic Oncologist, 05. General Practitioner/Internist, 06. Plastic Surgeon/Reconstructive Surgeon, 07. Medical Oncologist, 08. Radiation Oncologist, 09. Urologist, 10. Other, 77. Don’t know / Not sure, 99. Refused
M14.06	Did any doctor, nurse, or other health professional ever give you a written summary of all the cancer treatments that you received?	1. Yes, 2. No, 7. Don’t know / Not sure, 9. Refused
M14.07	Have you ever received instructions from a doctor, nurse, or other health professional about where you should return or who you should see for routine cancer checkups after completing your treatment for cancer?	1. Yes, 2. No, 7. Don’t know / Not sure, 9. Refused
M14.08	Were these instructions written down or printed on paper for you?	1. Yes, 2. No, 7. Don’t know / Not sure, 9. Refused
M14.09	With your most recent diagnosis of cancer, did you have health insurance that paid for all or part of your cancer treatment?	1. Yes, 2. No, 7. Don’t know / Not sure, 9. Refused
M14.10	Were you ever denied health insurance or life insurance coverage because of your cancer?	1. Yes, 2. No, 7. Don’t know / Not sure, 9. Refused
M14.11	Did you participate in a clinical trial as part of your cancer treatment?	1. Yes, 2. No, 7. Don’t know / Not sure, 9. Refused
M14.12	Do you currently have physical pain caused by your cancer or cancer treatment?	1. Yes, 2. No, 7. Don’t know / Not sure, 9. Refused
M14.13	Would you say your pain is currently under control…?	1. With medication (or treatment), 2. Without medication (or treatment), 3. Not under control, with medication (or treatment), 4. Not under control, without medication (or treatment), 7. Don’t know / Not sure, 9. Refused
M15.01	Which one of the following best describes the decision to have the P.S.A. test done?	1. You made the decision alone, 2. Your doctor, nurse, or health care provider made the decision alone, 3. You and one or more other persons made the decision together, 4. You don’t know how the decision was made, 9. Refused
M15.02	Who made the decision with you?	1. Doctor/nurse/health care provider, 2. Spouse/significant other, 3. Other family member, 4. Friend/nonrelative, 7. Don’t know / Not sure, 9. Refused
M16.01	A clinical breast exam is when a doctor, nurse, or other health professional feels the breasts for lumps. Have you ever had a clinical breast exam?	1. Yes, 2. No, 7. Don’t know/ not sure, 9. Refused
M16.02	How long has it been since your last breast exam?	1. Within the past year (anytime less than 12 months ago), 2. Within the past 2 years (1 year but less than 2 years ago), 3. Within the past 3 years (2 years but less than 3 years ago), 4. Within the past 5 years (3 years but less than 5 years ago), 5. 5 or more years ago, 7. Don’t know / Not sure, 9. Refused
M17.01	A vaccine to prevent the human papillomavirus or H.P.V. infection is available and is called the cervical cancer or genital warts vaccine, H.P.V. shot, [Fill: if female GARDASIL or CERVARIX; if male: GARDASIL]. Have you ever had an H.P.V. vaccination?	1. Yes, 2. No, 3. Doctor refused when asked, 7. Don’t know/ not sure, 9. Refused
M17.02	How many H.P.V. shots did you receive?	00. Number of shots, 03. All shots, 77. Don’t know / Not sure, 99. Refused
M18.01	Have you received a tetanus shot in the past 10 years?	1. Yes, received Tdap, 2. Yes, received tetanus shot, but not Tdap, 3. Yes, received tetanus shot but not sure what type, 4. No, did not receive any tetanus shot in the past 10 years, 7. Don’t know/Not sure, 9. Refused
M19.01	Have you ever had the shingles or zoster vaccine?	1. Yes, 2. No, 7. Don’t know/ not sure, 9. Refused
M20.01	What kind of work do you do? For example, registered nurse, janitor, cashier, auto mechanic.	Open response or 99. Refused
M20.02	What kind of business or industry do you work in? For example, hospital, elementary school, clothing manufacturing, restaurant.	Open response or 99. Refused
M21.01a	Which of the following best represents how you think of yourself?	1. Gay, 2. Straight, that is, not gay, 3. Bisexual, 4. Something else, 7. I don't know the answer, 9. Refused
M21.01b	Which of the following best represents how you think of yourself?	1. Lesbian or Gay, 2. Straight, that is, not gay, 3. Bisexual, 4. Something else, 7. I don't know the answer, 9. Refused
M21.02	Do you consider yourself to be transgender?	1. Yes, Transgender, male-to-female, 2. Yes, Transgender, female to male, 3. Yes, Transgender, gender nonconforming, 4. No, 7. Don’t know/not sure, 9. Refused
M22.01	What is the birth month and year of the [Xth] child?	Month/Year or 77/7777 Don’t know / Not sure, 99/9999 Refused
M22.02	Is the child a boy or a girl?	1. Boy, 2. Girl, 9. Refused
M22.03	Is the child Hispanic, Latino/a, or Spanish origin?	1. Mexican, Mexican American, Chicano/a, 2. Puerto Rican, 3. Cuban, 4. Another Hispanic, Latino/a, or Spanish origin, 5. No, 7. Don’t know / Not sure, 9. Refused
M22.04	Which one or more of the following would you say is the race of the child?	10. White, 20. Black or African American, 30. American Indian or Alaska Native, 40. Asian, 41. Asian Indian, 42. Chinese, 43. Filipino, 44. Japanese, 45. Korean, 46. Vietnamese, 47. Other Asian, 50. Pacific Islander, 51. Native Hawaiian, 52. Guamanian or Chamorro, 53. Samoan, 54. Other Pacific Islander, 60. Other, 77. Don’t know / Not sure, 99. Refused
M22.05	Which one of these groups would you say best represents the child’s race?	10. White, 20. Black or African American, 30. American Indian or Alaska Native, 40. Asian, 41. Asian Indian, 42. Chinese, 43. Filipino, 44. Japanese, 45. Korean, 46. Vietnamese, 47. Other Asian, 50. Pacific Islander, 51. Native Hawaiian, 52. Guamanian or Chamorro, 53. Samoan, 54. Other Pacific Islander, 60. Other, 88. No additional choices, 77. Don’t know / Not sure, 99. Refused
M22.06	How are you related to the child? Are you a….	1. Parent (include biologic, step, or adoptive parent), 2. Grandparent, 3. Foster parent or guardian, 4. Sibling (include biologic, step, and adoptive sibling), 5. Other relative, 6. Not related in any way, 7. Don’t know / Not sure, 9. Refused
M23.01	The next two questions are about the Xth child. Has a doctor, nurse or other health professional EVER said that the child has asthma?	1. Yes, 2. No, 7. Don’t know/ not sure, 9. Refused
M23.02	Does the child still have asthma?	1. Yes, 2. No, 7. Don’t know/ not sure, 9. Refused
CB01.01	Would it be okay if we called you back to ask additional asthma related questions at a later time?	1. Yes, 2. No
CB01.02	Which person in the household was selected as the focus of the asthma call-back?	1. Adult, 2. Child
CB02.01	We would like to call you again to talk in more detail about your reproductive health. The information will be used to help develop and improve the programs in . Would it be okay if we called you back to ask questions related to reproductive health at a later time?	1. Yes, 2. No

**Table 6:** 2018 Behavioral Risk Surveillance System/Asthma Call Back Survey - Adult Questionnaire

Question No.	Question Text	Response Options
1.1	Are you {sample person’s name}?	1. Yes (go to informed consent), 2. No
1.2	May I speak with {sample person’s name}?	1. Yes, 2. No
1.3	Enter time/date for return call	Free text
1.4	Hello, my name is ____. I’m calling on behalf of the {STATE NAME} health department...	N/A
2.1	Have you ever been told by a doctor or other health professional that you have asthma?	1. Yes, 2. No, 7. Don't know, 9. Refused
2.2	Do you still have asthma?	1. Yes, 2. No, 7. Don't know, 9. Refused
2.3	May we combine your answers to this survey with your answers from the survey you did a few weeks ago?	1. Yes, 2. No, 7. Don't know, 9. Refused
3.1	How old were you when you were first told by a doctor or other health professional that you had asthma?	__ __ (Enter age in years)
3.2	How long ago was that? Was it...	1. Within the past 12 months, 2. 1-5 years ago, 3. More than 5 years ago, 7. Don't know, 9. Refused
3.3	How long has it been since you last talked to a doctor or other health professional about your asthma?	88. Never, 04. Within the past year, 05. 1 year to less than 3 years ago, 06. 3 years to 5 years ago, 07. More than 5 years ago, 77. Don't know, 99. Refused
3.4	How long has it been since you last took asthma medication?	88. Never, 01. Less than one day ago, 02. 1-6 days ago, 03. 1 week to less than 3 months ago, 04. 3 months to less than 1 year ago, 05. 1 year to less than 3 years ago, 06. 3 years to 5 years ago, 07. More than 5 years ago, 77. Don't know, 99. Refused
3.5	How long has it been since you last had any symptoms of asthma?	88. Never, 01. Less than one day ago, 02. 1-6 days ago, 03. 1 week to less than 3 months ago, 04. 3 months to less than 1 year ago, 05. 1 year to less than 3 years ago, 06. 3 years to 5 years ago, 07. More than 5 years ago, 77. Don't know, 99. Refused
4.1	During the past 30 days, on how many days did you have any symptoms of asthma?	__ __ Days (1-29), 88. No symptoms, 30. Every day, 77. Don't know, 99. Refused
4.2	Do you have symptoms all the time? “All the time” means symptoms that continue throughout the day. It does not mean symptoms for a little while each day.	1. Yes, 2. No, 7. Don't know, 9. Refused
4.3	During the past 30 days, on how many days did symptoms of asthma make it difficult for you to stay asleep?	__ __ Days/nights, 88. No symptoms, 30. Every day, 77. Don't know, 99. Refused
4.4	During the past two weeks, on how many days were you completely symptom-free, that is no coughing, wheezing, or other symptoms of asthma?	__ __ Number of days, 88. None, 77. Don't know, 99. Refused
4.5	During the past 12 months, have you had an episode of asthma or an asthma attack?	1. Yes, 2. No, 7. Don't know, 9. Refused
4.6	During the past three months, how many asthma episodes or attacks have you had?	__ __ None, 888. None, 777. Don't know, 999. Refused
4.7	How long did your MOST RECENT asthma episode or attack last?	1. __ __ Minutes, 2. __ __ Hours, 3.__ __ Days, 4.__ __ Weeks, 555. Never, 777, Don’t know/Not Sure, 999. Refused
4.8	Compared with other episodes or attacks, was this most recent attack shorter, longer, or about the same?	1. Shorter, 2. Longer, 3. About the Same, 4. The Most Recent Attack was Actually the First Attack, 7. Don’t Know, 9. Refused
5.01	Do you have any kind of health care coverage, including health insurance, prepaid plans such as HMOs, or government plans such as Medicare or Medicaid?	1. Yes, 2. No, 7. Don't know, 9. Refused
5.02	During the past 12 months was there any time that you did not have any health insurance or coverage?	1. Yes, 2. No, 7. Don't know, 9. Refused
5.1	During the past 12 months how many times did you see a doctor or other health professional for a routine checkup for your asthma?	__ __ __ (Enter number), 888. None, 777. Don't know, 999. Refused
5.2	An urgent care center treats people with illnesses or injuries that must be addressed immediately and cannot wait for a regular medical appointment. During the past 12 months, have you had to visit an emergency room or urgent care center because of your asthma?	1. Yes, 2. No, 7. Don't know, 9. Refused
5.3	During the past 12 months, how many times did you visit an emergency room or urgent care center because of your asthma?	__ __ __ (Enter number), 888. None, 777. Don't know, 999. Refused
5.4	During the past 12 months, how many times did you see a doctor or other health professional for urgent treatment of worsening asthma symptoms or for an asthma episode or attack?	__ __ __ (Enter number), 888. None, 777. Don't know, 999. Refused
5.5	During the past 12 months, that is since [1 YEAR AGO TODAY], have you had to stay overnight in a hospital because of your asthma? Do not include an overnight stay in the emergency room.	1. Yes, 2. No, 7. Don't know, 9. Refused
5.6A	During the past 12 months, how many different times did you stay in any hospital overnight or longer because of your asthma?	__ __ __ Times, 777. Don't know, 999. Refused
5.7	The last time you left the hospital, did a health professional TALK with you about how to prevent serious attacks in the future?	1. Yes, 2. No, 7. Don't know, 9. Refused
5.8A	During the past 12 months, how many days were you unable to work or carry out your usual activities because of your asthma?	__ __ __ (Enter Number of Days), 888. Zero, 777. Don’t know, 999. Refused
5.9	During just the past 30 days, would you say you limited your usual activities due to asthma not at all, a little, a moderate amount, or a lot?	1. Not at all, 2. A Little, 3. A Moderate Amount, 4. A Lot, 7. Don't know, 9. Refused
6.1	Has a doctor or other health professional ever taught you how to recognize early signs or symptoms of an asthma episode?	1. Yes, 2. No, 7. Don't know, 9. Refused
6.2	Has a doctor or other health professional ever taught you what to do during an asthma episode or attack?	1. Yes, 2. No, 7. Don't know, 9. Refused
6.3	A peak flow meter is a hand held device that measures how quickly you can blow air out of your lungs. Has a doctor or other health professional ever taught you how to use a peak flow meter to adjust your daily medications?	1. Yes, 2. No, 7. Don't know, 9. Refused
6.4	Has a doctor or other health professional EVER given you an asthma action plan?	1. Yes, 2. No, 7. Don't know, 9. Refused
6.5	Have you ever taken a course or class on how to manage your asthma?	1. Yes, 2. No, 7. Don't know, 9. Refused
7.1	Is an air cleaner or purifier regularly used inside your home?	1. Yes, 2. No, 7. Don't know, 9. Refused
7.2	Is a dehumidifier regularly used to reduce moisture inside your home?	1. Yes, 2. No, 7. Don't know, 9. Refused
7.3	Is an exhaust fan that vents to the outside used regularly when cooking in your kitchen?	1. Yes, 2. No, 7. Don't know, 9. Refused
7.4	Is gas used for cooking?	1. Yes, 2. No, 7. Don't know, 9. Refused
7.5	In the past 30 days, has anyone seen or smelled mold or a musty odor inside your home? Do not include mold on food.	1. Yes, 2. No, 7. Don't know, 9. Refused
7.6	Does your household have pets such as dogs, cats, hamsters, birds or other feathered or furry pets that spend time indoors?	1. Yes, 2. No, 7. Don't know, 9. Refused
7.7	Are pets allowed in your bedroom?	1. Yes, 2. No, 7. Don't know, 9. Refused
7.8	In the past 30 days, has anyone seen a cockroach inside your home?	1. Yes, 2. No, 7. Don't know, 9. Refused
7.9	In the past 30 days, has anyone seen mice or rats inside your home? Do not include mice or rats kept as pets.	1. Yes, 2. No, 7. Don't know, 9. Refused
7.10	Is a wood burning fireplace or wood burning stove used in your home?	1. Yes, 2. No, 7. Don't know, 9. Refused
7.11	Are unvented gas logs, unvented gas fireplaces, or unvented gas stoves used in your home?	1. Yes, 2. No, 7. Don't know, 9. Refused
7.12	In the past week, has anyone smoked inside your home?	1. Yes, 2. No, 7. Don't know, 9. Refused
7.13	Has a health professional ever advised you to change things in your home, school, or work to improve your asthma?	1. Yes, 2. No, 7. Don't know, 9. Refused
7.14	Do you use a mattress cover that is made especially for controlling dust mites?	1. Yes, 2. No, 7. Don't know, 9. Refused
7.15	Do you use a pillow cover that is made especially for controlling dust mites?	1. Yes, 2. No, 7. Don't know, 9. Refused
7.16	Do you have carpeting or rugs in your bedroom? This does not include throw rugs small enough to be laundered.	1. Yes, 2. No, 7. Don't know, 9. Refused
7.17	Are your sheets and pillowcases washed in cold, warm, or hot water?	1. Cold, 2. Warm, 3. Hot, 4. Varies, 7. Don't know, 9. Refused
7.18	In your bathroom, do you regularly use an exhaust fan that vents to the outside?	1. Yes, 2. No or “No Fan”, 7. Don't know, 9. Refused
8.1	Have you ever used over-the-counter medication for your asthma?	1. Yes, 2. No, 7. Don't know, 9. Refused
8.2	Have you ever used a prescription inhaler?	1. Yes, 2. No, 7. Don't know, 9. Refused
8.3	Did a doctor or other health professional show you how to use the inhaler?	1. Yes, 2. No, 7. Don't know, 9. Refused
8.4	Did a doctor or other health professional watch you use the inhaler?	1. Yes, 2. No, 7. Don't know, 9. Refused
8.5	Can you please go get the asthma medicines while I wait on the phone?	1. Yes, 2. No, 3. Respondent knows the meds, 7. Don't know, 9. Refused
8.7	Do you all the medications?	1. Yes I have all the medications, 2. Yes I have some of the medications but not all, 7. Don't know, 9. Refused
8.8	In the past 3 months have you taken prescription asthma medicine using an inhaler?	1. Yes, 2. No, 7. Don't know, 9. Refused
8.9	In the past 3 months, what prescription asthma medications did you take by inhaler?	01. Advair (Advair Diskus), 02. Aerobid, 03. Albuterol (Albuterol sulfate or salbutamol), 04. Alupent, 43. Alvesco (Ciclesonide), 40. Asmanex (twisthaler), 05. Atrovent, 06. Azmacort, 07. Beclomethasone dipropionate, 08. Beclovent, 09. Bitolterol, 11. Budesonide, 12. Combivent, 13. Cromolyn, 44. Dulera, 14. Flovent, 15. Flovent Rotadisk, 16. Flunisolide, 17. Fluticasone, 34. Foradil, 35. Formoterol, 19. Ipratropium Bromide, 37. Levalbuterol tartrate, 20. Maxair, 21. Metaproteronol, 39. Mometasone furoate, 22. Nedocromil, 23. Pirbuterol, 41. Pro-Air HFA, 24. Proventil, 25. Pulmicort Flexhaler, 36. QVAR, 26. Salmeterol, 27. Serevent, 42. Symbicort, 28. Terbutaline (Terbutaline sulfate), 30. Tornalate, 31. Triamcinolone acetonide, 32. Vanceril, 33. Ventolin, 38. Xopenex HFA, 66. Other, Please Specify, 88. No prescription inhalers, 77. Don’t know, 99. Refused
8.10	Enter other medication from (8.9) in text field if more than one medication is given, enter all medications on one line.	
8.13	A spacer is a small attachment for an inhaler that makes it easier to use. Do you use a spacer with [MEDICINE FROM INH_MEDS SERIES]?	1. Yes, 2. No, 3. Medication is a dry powder inhaler or disk inhaler, 4. Medication has a built-in spacer/does not need a spacer, 7. Don’t know, 9. Refused
8.14	In the past 3 months, did you take [MEDICINE FROM INH_MEDS SERIES] when you had an asthma episode or attack?	1. Yes, 2. No, 3. No attack in past 3 months, 7. Don’t know, 9. Refused
8.15	In the past 3 months, did you take [MEDICINE FROM INH_MEDS SERIES] before exercising?	1. Yes, 2. No, 3. Didn’t exercise in past 3 months, 7. Don’t know, 9. Refused
8.16	In the past 3 months, did you take [MEDICINE FROM INH_MEDS SERIES] on a regular schedule everyday?	1. Yes, 2. No, 7. Don’t know, 9. Refused
8.18	How many times per day or per week do you use [MEDICINE FROM INH_MEDS SERIES]?	3 _ _ Times per day, 4 _ _ Times per week, 5. Never, 6. Less often than once a week, 7. Don’t know / Not sure, 9. Refused
8.19	How many canisters of [MEDICINE FROM INH_MEDS SERIES] have you used in the past 3 months?	___ Canisters, 77. Don’t know, 88. None, 99. Refused
8.20	In the past 3 months, have you taken any prescription medicine in pill form for your asthma?	1. Yes, 2. No, 7. Don't know, 9. Refused
8.21	What prescription asthma medications do you take in pill form?	1. Accolate, 02. Aerolate, 03. Albuterol, 04. Alupent, 49. Brethine, 05. Choledyl (oxtriphylline), 07. Deltasone, 08. Elixophyllin, 11. Medrol, 12. Metaprel, 13. Metaproteronol, 14. Methylprednisolone, 15. Montelukast, 17. Pediapred, 18. Prednisolone, 19. Prednisone, 21. Proventil, 23. Respid, 24. Singulair, 25. Slo-phyllin, 26. Slo-bid, 48. Terbutaline (T. sulfate), 28. Theo-24, 30. Theochron, 31. Theoclear, 32. Theodur, 33. Theo-Dur, 35. Theophylline, 37. Theospan, 40. T-Phyl, 42. Uniphyl, 43. Ventolin, 44. Volmax, 45. Zafirlukast, 46. Zileuton, 47. Zyflo Filmtab, 66. Other, please specify, 88. No pills, 77. Don’t Know, 99. Refused
8.22	In the past 3 months, did you take [MEDICATION LISTED IN PILLS_MD] on a regular schedule every day?	1. Yes, 2. No, 7. Don't know, 9. Refused
8.23	In the past 3 months, have you taken any prescription asthma medication in syrup form?	1. Yes, 2. No, 7. Don't know, 9. Refused
8.24	What prescription asthma medications have you taken as a syrup?	1. Aerolate, 02. Albuterol, 03. Alupent, 04. Metaproteronol, 05. Prednisolone, 06. Prelone, 07. Proventil, 08. Slo-Phyllin, 09. Theophyllin, 10. Ventolin, 66. Other, please specify, 88. No Syrups, 77. Don’t know, 99. Refused
8.25	In the past 3 months, were any of your prescription asthma medicines used with a nebulizer?	1. Yes, 2. No, 7. Don't know, 9. Refused
8.26a	In the past 3 months did you use a nebulizer at home?	1. Yes, 2. No, 7. Don't know, 9. Refused
8.26b	In the past 3 months did you use a nebulizer at a doctor’s office?	1. Yes, 2. No, 7. Don't know, 9. Refused
8.26c	In the past 3 months did you use a nebulizer in an Emergency Room?	1. Yes, 2. No, 7. Don't know, 9. Refused
8.26d	In the past 3 months did you use a nebulizer at work or at school?	1. Yes, 2. No, 7. Don't know, 9. Refused
8.26e	In the past 3 months did you use a nebulizer at any other place?	1. Yes, 2. No, 7. Don't know, 9. Refused
8.27	In the past 3 months, what prescriptions asthma medications have you taken using a nebulizer?	1. Albuterol, 02. Alupent, 03. Atrovent, 04. Bitolterol, 05. Budesonide, 17. Combivent, 06. Cromolyn, 07. DuoNeb, 08. Intal, 09. Ipratropium bromide, 10. Levalbuterol, 11. Metaproteronol, 18. Perforomist, 12. Proventil, 13. Pulmicort, 14. Tornalate, 15. Ventolin, 16. Xopenex, 66. Other, please specify, 88. No Nebulizers, 77. Don’t Know, 99. Refused
8.28	In the past 3 months, did you take [MEDICINE FROM NEB_ID SERIES] when you had an asthma episode or attack?	1. Yes, 2. No, 3. No attack in past 3 months, 7. Don’t know, 9. Refused
8.29	In the past 3 months, did you take [MEDICINE FROM NEB_ID SERIES] on a regular schedule every day?	1. Yes, 2. No, 7. Don’t know, 9. Refused
8.30	How many times per day or per week do you use [MEDICINE FROM NEB_ID SERIES]?	3. _ _ Days, 4 _ _ Weeks, 555. Never, 666. Less often than once a week, 777. Don’t know / Not sure, 999. Refused
9.1	Was there a time in the past 12 months when you needed to see your primary care doctor for your asthma but could not because of the cost?	1. Yes, 2. No, 7. Don't know, 9. Refused
9.3	Was there a time in the past 12 months when you needed to buy medication for your asthma but could not because of the cost?	1. Yes, 2. No, 7. Don't know, 9. Refused
10.1	How would you describe your current employment status?	1. Employed full-time, 2. Employed part-time, 3. Not employed, 7. Don't know, 9. Refused
10.2	What is the main reason you are not now employed?	01. Keeping house, 02. Going to school, 03. Retired, 04. Disabled, 05. Unable to work for other health reasons, 06. Looking for work, 07. Laid off, 08. Other, 77. Don’t know, 99. Refused
10.3	Have you ever been employed?	1. Yes, 2. No, 7. Don’t know, 9. Refused
10.4	Are your asthma symptoms MADE WORSE by things like chemicals, smoke, dust or mold in your CURRENT job?	1. Yes, 2. No, 7. Don’t know, 9. Refused
10.5	Was your asthma first CAUSED by things like chemicals, smoke, dust or mold in your CURRENT job?	1. Yes, 2. No, 7. Don’t know, 9. Refused
10.6	Was your asthma first CAUSED by things like chemicals, smoke, dust or mold in any PREVIOUS job you ever had?	1. Yes, 2. No, 7. Don’t know, 9. Refused
10.7	Did you ever lose or quit a job because things in the workplace, like chemicals, smoke, dust or mold, caused your asthma or made your asthma symptoms worse?	1. Yes, 2. No, 7. Don’t know, 9. Refused
10.8	Were your asthma symptoms MADE WORSE by things like chemicals, smoke, dust or mold in any PREVIOUS job you ever had?	1. Yes, 2. No, 7. Don’t know, 9. Refused
10.9	Did you and a doctor or other health professional ever discuss whether your asthma could have been caused by, or made worse by, any job you ever had?	1. Yes, 2. No, 7. Don't know, 9. Refused
10.10	Have you ever been TOLD BY a doctor or other health professional that your asthma was caused by, or your symptoms made worse by, any job you ever had?	1. Yes, 2. No, 7. Don't know, 9. Refused
10.11	Have YOU ever TOLD a doctor or other health professional that your asthma was caused by, or your symptoms made worse by, any job you ever had?	1. Yes, 2. No, 7. Don't know, 9. Refused
11.1	Have you ever been told by a doctor or health professional that you have chronic obstructive pulmonary disease (COPD)?	1. Yes, 2. No, 7. Don't know, 9. Refused
11.2	Have you ever been told by a doctor or other health professional that you have emphysema?	1. Yes, 2. No, 7. Don't know, 9. Refused
11.3	Have you ever been told by a doctor or other health professional that you have Chronic Bronchitis?	1. Yes, 2. No, 7. Don't know, 9. Refused
11.4	Have you ever been told by a doctor or other health professional that you were depressed?	1. Yes, 2. No, 7. Don't know, 9. Refused
12.1	In the past 12 months, have you used herbs to control your asthma?	1. Yes, 2. No, 7. Don't know, 9. Refused
12.2	In the past 12 months, have you used vitamins to control your asthma?	1. Yes, 2. No, 7. Don't know, 9. Refused
12.3	In the past 12 months, have you used acupuncture to control your asthma?	1. Yes, 2. No, 7. Don't know, 9. Refused
12.4	In the past 12 months, have you used acupressure to control your asthma?	1. Yes, 2. No, 7. Don't know, 9. Refused
12.5	In the past 12 months, have you used aromatherapy to control your asthma?	1. Yes, 2. No, 7. Don't know, 9. Refused
12.6	In the past 12 months, have you used homeopathy to control your asthma?	1. Yes, 2. No, 7. Don't know, 9. Refused
12.7	In the past 12 months, have you used reflexology to control your asthma?	1. Yes, 2. No, 7. Don't know, 9. Refused
12.8	In the past 12 months, have you used yoga to control your asthma?	1. Yes, 2. No, 7. Don't know, 9. Refused
12.9	In the past 12 months, have you used breathing techniques to control your asthma?	1. Yes, 2. No, 7. Don't know, 9. Refused
12.10	In the past 12 months, have you used naturopathy to control your asthma?	1. Yes, 2. No, 7. Don't know, 9. Refused
12.11	Besides the types I have just asked about, have you used any other type of alternative care for your asthma in the past 12 months?	1. Yes, 2. No, 7. Don't know, 9. Refused
12.13	What else have you used?	
